# Strengthening national health research systems in the WHO African Region – progress towards universal health coverage

**DOI:** 10.1186/s12992-019-0492-8

**Published:** 2019-07-26

**Authors:** Simbarashe Rusakaniko, Michael Makanga, Martin O. Ota, Moses Bockarie, Geoffrey Banda, Joseph Okeibunor, Francisca Mutapi, Prosper Tumusiime, Thomas Nyirenda, Joses Muthuri Kirigia, Juliet Nabyonga-Orem

**Affiliations:** 1 Department of Community Medicine, College of Health Sciences, University of Zimbabwe, PO Box A178, Avondale, Harare, Zimbabwe; 2 The European & Developing Countries Clinical Trials Partnership (EDCTP), Anna van Saksenlaan 51, 2593 HW The Hague, The Netherlands; 3 GSK Building WN23, Avenue Fleming 20, 1300 Wavre, Belgium; 4 Health Systems and Services cluster, World Health Organization Regional Office for Africa, Cite de Djoue, BP 06 Brazzaville, Republic of the Congo; 5 Institute of Immunology & Infection Research, University of Edinburgh, Ashworth Laboratories, King’s Buildings, Charlotte Auerbach Road, Edinburgh, EH9 3FL UK; 6 Independent consultant, Nairobi, Kenya; 7 Inter-Country Support Team for Eastern & Southern Africa, Health Systems and Services Cluster, World Health Organization, P.O Box CY 348, Causeway, Harare, Zimbabwe; 8 TIBA Partnership, NIHR Global Health Research Unit Tackling Infections to Benefit Africa (TIBA), University of Edinburgh, Edinburgh, UK

**Keywords:** Barometer, National health research systems, Research for health governance, Financing for research, Research coordination

## Abstract

**Background:**

Health challenges and health systems set-ups differ, warranting contextualised healthcare interventions to move towards universal health coverage. As such, there is emphasis on generation of contextualized evidence to solve local challenges. However, weak research capacity and inadequate resources remain an impendiment to quality research in the African region. WHO African Region (WHO AFR) facilitated the adoption of a regional strategy for strengthening national health research systems (NHRS) in 2015. We assessed the progress in strengthening NHRS among the 47 member states of the WHO AFR.

**Methods:**

We employed a cross sectional survey design using a semi structured questionnaire. All the 47member states of WHO AFR were surveyed. We assessed performance against indicators of the regional research strategy, explored facilitating factors and barriers to strengthening NHRS. Using the research barometer, which is a metric developed for the WHO AFR we assessed the strength of NHRS of member states. Data were analysed in Excel Software to calculate barometer scores for NHRS function and sub-function. Thematic content was employed in analysing the qualitative data. Data for 2014 were compared to 2018 to assess progress.

**Results:**

WHO AFR member states have made significant progress in strengthening their NHRS. Some of the indicators have either attained or exceeded the 2025 targets. The average regional barometer score improved from 43% in 2014 to 61% in 2018. Significant improvements were registered in the governance of research for health (R4H); developing and sustaining research resources and producing and using research. Financing R4H improved only modestly. Among the constraints are the lengthy ethical clearance processes, weak research coordination mechanisms, weak enforcement of research laws and regulation, inadequate research infrastructure, limited resource mobilisation skills and donor dependence.

**Conclusion:**

There has been significant improvement in the NHRS of member states of the WHO AFRO since the last assessment in 2014. Improvement across the different objectives of the regional research strategy is however varied which compromises overall performance. The survey highlighted the areas with slow improvement that require a concerted effort. Furthermore, the study provides an opportunity for countries to share best practice in areas of excellence.

## Introduction

The United Nations’ Sustainable Development Goal (SDG) 3 commits governments around the world to ensure health security and promote well-being for all, at all ages. Central to the health SDG is the Universal Health Coverage (UHC) target, which seeks to ensure that all people have access to quality, effective and affordable health services [1]. African countries however face a number of challenges chief among which are limited resources, the double burden of communicable and non-communicable diseases [2, 3], new challenges such as climate change, in addition to demographic and epidemiologic transitions. Systemic and investment challenges persisit including how to ensure health equity, affordably scale up and deliver innovative and sustainable healthcare to populations in need.

Current projections in the 2017 global burden of diseases study [4] indicate that many health-related SDG indicators will require a rethinking from curative interventions towards multisectoral activities by national disease control programmes focusing on prevention-oriented policy action, and more national investments. Given the climatic, economic, social and disease epidemiological diversity on the continent, health care challenges and health systems set-ups invariably differ and therefore require contextualised healthcare interventions. This complexity accentuates the importance of strengthening national health research systems that generate timely knowledge and innovations to address local health challenges and progress towards UHC and health security for countries. Research can unlock the drivers of health in the African Region, support the discovery, design and delivery of effective interventions to address current health issues and eliminate inequities in access to health, as well as lay the foundation for preventing poor health in future generations [5]. Failure to prioritize local research will result in the roots and triggers of poor health in Africa being misinterpreted, the soundest interventions for addressing them unarticulated, the strategies for optimizing the effectiveness of health actions remaining elusive and SDG3 remaining a mirage.

The World Health Report 2013 [1], emphasizes the importance of supporting the health research community within countries and worldwide. The report highlights three themes: Universal Health Coverage; conducting and using research; and systems approaches developed locally. The national health research systems (NHRS) facilitate the generation and utilization of scientific knowledge and innovations for developing technologies, as well as systems and services to achieve UHC. A survey of the NHRS of the members states of the WHO African Region in 2014 highlighted the African Region’s low contribution to global health research; the weak capacity for health research; the low priority accorded to research as a tool for solving the region’s health needs and; limited use of research evidence in decision making. Effort to mitigate this resulted in the development of Research for Health: a Strategy for The African Region, 2016–2025, which was endorsed at the 65th session of the WHO Regional Committee for Africa [6]. It covers four NHRS functions namely: Governance of research for health (R4H), Developing and sustaining resources for R4H, Producing and using R4H, and Financing of R4H. Implementation of this strategy has been ongoing in member states since its endorsement in 2015. A NHRS barometer was developed to assess the performance of NHRS functions of member states in the WHO African Region using a set of criteria [7, 8]. The NHRS scores for the individual sub-functions provide a metric that can guide policymakers to strategically allocate resources to poor performance areas.

This study assessed the progress towards strengthening the NHRSs among the 47 member states of the WHO African Region (WHO AFR) as compared to baseline of 2014.

## Methodology

We employed a cross sectional survey design using a semi structured mailed questionnaire. All the 47 member states of WHO AFR were included in the survey. The assessment was undertaken in line with the objectives of the regional health research strategy [6]. We assessed performance against indicators in the research strategy and collected data on presence/attainment of a given indicator or the absence thereof – See Table 1. We sought perceptions of respondents on facilitating factors as well as barriers to strengthening NHRS, and also asked them to rank the level of importance of the different sources of financing R4H.

**Table 1:** Indicators assessed under the four different objectives

Objective	Indicators
Strengthening research Governance	• Countries with valid health research policies, strategic plans, and priority lists • Countries with legislation on R4H • All countries with national or institutional ethics review committees • At least 80% of countries have a national or institutional ethics review committee assessing & providing feedback within 3 months.
Creating & sustaining resources	• Countries with a health research promoting unit within the MOH • Countries with universities/colleges that have a training programme in health research • Countries with a national health research institute/council
Producing and using health research	• Countries with a R & D coordination mechanism • Each country to increase the number of articles published in peer reviewed journals byat least 30%. • Countries with a knowledge translation platform
Financing health research	• Countries that have a dedicated budget line for R4H • Countries investing at least 2% of the national health budget in R4H • Countries investing at least 5% of health sector development assistance in R4H • Countries regularly tracking R4H spending from all sources

Source: WHO/AFRO [6]

Data were collected between December 2017 – August 2018. Prior to data collection, a meeting was convened for the heads of national health research coordination institutions, focal points for research in ministries of health and a representative of institutions undertaking research in the country to orient them on the methodology and the questionnaire. The questionnaire was primarily filled in by the ministry of health focal point for research in the country or the head of national health research coordination institute as the arrangements differed in the different countries. The completed questionnaire was validated by an in country team comprised of representatives of institutions conducting health research, the head of the national research coordination institution, focal point for research in the WHO Country office and the focal point for research in the ministry of health.

### Data analysis

Data were analysed in Excel Software to assess performance against the different indicators and, calculate NHRS function and sub-function indices (barometer scores).

The assessment of performance against indicators entailed counting the number of countries that responded ‘yes’ indicating presence or ‘no’ indicating absence. In assessing the level of importance of the different sources of financing for R4H, an average score for each element (Government Revenue, Private Sector Companies, Multilateral and Bilateral Donors, Local NGOs and International NGOs) was computed across all the countries that responded and presented in a radar diagram.

In computing barometer scores, we followed the previously used method in the WHO African Region (AFR) [8]. The NHRS barometer analysed performance against the objectives of the research strategy for the WHO AFR [6]. It covers four NHRS functions namely: *Governance of research for health (R4H), Developing and sustaining resources for R4H, Producing and using R4H, and Financing of R4H* which have been described elsewhere [8].

There are sub functions under each NHRS function as shown in Table 2. The choice of sub functions is derived from previous work on assessing NHRS by Kirigia et al. [8].

**Table 2:** National Health Research Systems sub-functions

Health research system barometer parameters	
*A. Governance of research for health*	
1. Health research policy index (RHRPI)	
2. Health research law index (RHRLI)	
3. Strategic health research plan index (RSHRPI)	
4. Ethical review committee index (RERCI)	
5. Health research priority list index (RHRPLI)	
6. Health research focal point index (RHRFPI	
*B. Developing and sustaining resources for R4H*	
7. Universities with faculties of health sciences/medicine (RUFHSI)	
8. Health research institutes or council (RHRCI)	
9. R4H programme (RHRPRI)	
10. R4H programme staff density index (RHRHRI)	
11. NGOs undertaking R4H index (RNGOI)	
*C. Producing and using research*	
12. R4H programme action plan index (RHRPAI)	
13. Knowledge translation platform index (RKTPI)	
14. Health research management forum index (RHRMFI)	
15. R4H publications per 100,000 population index (RPPCI)	
*D. Financing of R4H*	
16. Budget line for R4H index (RBLHRI)	
17. Government spending on R4H index (RHRBI)	

The methodology entailed calculation of sub-function indicies, each of the sub function index was calculated using the formula:

$$ Sub\  Function\ Index=\left(\frac{Actual\ score\  xi- Minimum\  xi\  Score}{Maximum\  xi\  Score- Minimum\  xi\  Score}\right), $$

where x_i_ is the i^th^ sub-function [8].

Sub functions were allocated a percentage score ranging from 0 to 100%. Dichotomous sub-functions with ‘yes’ or ‘no’ answer were allocated a 0% for non-existence and 100% for existence. Examples here include if a country has a research policy or not, has a prioritised research list or not. For sub-functions with continuous value answers, the score was the actual value. For example, for the number of technical and support staff sub-function question ‘How many technical and support staff are there in the programme?’ the actual value was the number of staff per 100,000 population, that is staff density. This value was obtained by dividing the total number of staff by the population and multiplying the outcome by 100,000.

To obtain the overall score for an individual country, average score was obtained across all the sub-functions that were reported in the country using the formula:

$$ {NHRSB}_{Score}=\left(\frac{\sum \limits_{i=1}^{17}{ SF I}_i}{TN_{SF}}\right) $$

where SFI is the sub-function index which is summed over the R4H sub-functions 1 to 17 and TN_SF_ is the total of all the sub-fuctions [8]. To obain the overall regional health reseach barometer, summation over all the regional health research barometer for each of the 17 sub-functions was obtained using the formula:

$$ {RHRSB}_{Score}=\left(\frac{\sum \limits_{i=1}^{17}{RSFI}_i}{TN_{SF}}\right), $$

where RSFI is the sub-function index which is summed over the R4H sub-functions 1 to 17 and TN_SF_ is the total of all the sub-fuctions [8].

The barometer scores for each country were calculated and the performance interpreted on a scale ranging from 0 to 100% [7]. NHRS barometer scores were categorised in conformity with the 2014 categories; as below average if less than 50%, average if the score was 50%, and above average if the score was over 50%.

To assess progress in strengthening NHRS, the difference between the two time points (2014 and 2018) was generated to determine if there has been any change.

Thematic analysis using manual coding was used to analyze the qualitative data, in line with the objectives of the study.

This study was a standing request by the Ministers of Health of the WHO African Region and the ethical approval for this 2018 survey of the NHRS was granted by the WHO African Regional Office’s Ethics Review Committee.

## Results

### Overall regional NHRS performance

Descriptive statistics show improvement in all the indicators under the different objectives of the research strategy compared to the 2014 baseline. In some cases, the 2025 targets of the Strategy have been met or surpassed – see Table 3. The 2025 targets for all indicators under “creating and sustaining resources”, except one, have been surpassed. The least progress is seen under the health financing objective with stagnation in all indicators except one (number of countries regularly tracking R4H spending from all sources). All indicators under the governance objective showed improvement but we also note that, both at baseline and in the 2018 survey, most of the indicators were already met by over 50% of countries.

**Table 3:** Summary of the Key indicators achievements in the four domain areas – Regional averages

	Baseline 2014 (*n* = 39) (% of countries)	Achievement 2018 (*n* = 39); (% of countries)	Target by 2025
Governance			
1. Countries with valid health research policies, strategic plans, and priority lists	20 (51%)	25 (65%)	100%.
2. Countries with legislation on R4H	15 (39%)	22 (56%)	80%.
3. All countries with national or institutional ethics review committees	36 (92%)	37 (95%)	100%.
4. At least 80% of countries have a national or institutional ethics review committee assessing & providing feedback within 3 months.	36 (92%)	37 (95%)	100%
Creating & sustaining resources			
1. Countries with a health research promoting unit within the MOH	16 (41%)	23 (59%)	75%.
2. Countries with universities/colleges that have a training programme in health research	36 (92%)	35 (90%)	40%.
3. Countries with a national health research institute/council	23 (59%)	28 (72%)	55%.
Producing and using health research			
1. Countries with a R & D coordination mechanism	28 (72%)	33 (85%)	85%.
2. Each country to increase the number of articles published in peer reviewed journals by at least 30%.	Not assessed	Not assessed	30%
3. Countries with a knowledge translation platform	16 (41%)	23 (59%)	100%.
Financing			
1. Countries that have a dedicated budget line for R4H	20 (51%)	24 (62%)	75%.
2. Countries investing at least 2% of the national health budget in R4H	1 (3%)	2 (8%) Cameroon/Mali	25%.
3. Countries investing at least 5% of health sector development assistance in R4H	1 (3%)	1 (4%) Cameroon	25%.
4. Countries regularly tracking R4H spending from all sources	34 (87%)	37 (95%)	50%.

Overall there was improvement in NHRS functions as evidenced by the Regional average research for health (R4H) barometer score (RHRSBScore) at 0.61 (61%) in 2018 compared to 0.40 (40%) in 2014 – See Table 4. There was improvement in barometer scores for all the NHRS sub functions. To understand the source of the poor or good scores, the performance of each NHRS function and its related sub-functions was further examined as summarized in Tables 3 and 4.

**Table 4:** Regional health research system barometer scores

Health research system barometer parameters	Regional barometer score		
	(2014) *n* = 39	(2018) *n* = 39	*p* value
*A. Governance of research for health*			
1. Regional health research policy index (RHRPI)	0.51	0.67	0.151
2. Regional health research law index (RHRLI)	0.38	0.56	0.111
3. Regional strategic health research plan index (RSHRPI)	0.51	0.49	0.860
4. Regional ethical review committee index (RERCI)	0.92	0.95	0. 591
5. Regional health research priority list index (RHRPLI)	0.59	0.79	0.028
6. Regional health research focal point index (RHRFPI)	0.82	0.85	0.361
*Average score for the governance of R4H*	0.62	0.72	0.174
*B. Developing and sustaining resources for R4H*			
7. Regional universities with faculties of health sciences/medicine (RUFHSI)	0.13	0.25	0.088
8. Regional health research institutes or council (RHRCI)	0.59	0.72	0.071
9. Regional R4H programme (RHRPRI)	0.56	0.72	0.071
10. Regional R4H programme staff density per 100,000 population index (RHRHRI)	0.001	0.002	0.909
11. Regional NGOs R4H index (RNGOI)	0.72	0.79	0.472
*Average score for developing and sustaining resources for R4H*	0.40	0.61	0.032
*C. Producing and using research*			
12. Regional R4H programme action plan index (RHRPAI)	0.51	0.59	0.478
13. Regional knowledge translation platform index (RKTPI)	0.40	0.59	0.047
14. Regional health research management forum index (RHRMFI)	0.38	0.46	0.474
15. Regional R4H publications per 100,000 population index (RPPCI)	0.10		
*Average score for producing and using research*	0.35	0.55	0.038
*D. Financing of R4H*			
16. Regional budget line for R4H index (RBLHRI)	0.51	0.62	0.164
17. Regional government spending on R4H index (RHRBI)	0.14	0.23	0.153
*Average score for financing of R4H*	0.33	0.43	0.182
*Regional health research systems barometer (RHRSB) average score*	0.43	0.61	0.056

### Establishing effective governance of research for health (R4H)

The Regional average barometer score for governance for R4H was above average in both surveys and shows improvement from 0.62 (62%) in 2014 to 0.72 (72%) in 2018. Significant improvement was noted in countries developing research priority lists (a barometer score of 79% in 2018 compared to 59% in 2014). Some indicators under governance performed better than others for example; the majority of countries had focal points for research in ministries of health and ethical review committees were in place in 95% of countries (2018 survey). Relatedly, the proportion of countries with research policies, strategies and legislation increased between 2014 and 2018 albeit marginally. Noteworthy is the significant number of countries that are still without policies and strategies (13) and prioritised research agendas (9).

#### Development of National Health Research Policy and National Health Research Strategic Plan

A number of countries are at various stages of developing their national health research policies. Sixty-five percent of the countries surveyed possess a national health research strategic plan however at varying levels ranging from expired, under development, extended to recently launched strategies. We observed improvement in the number of countries developing research priority lists. These activities demonstrate purposive and strategic policy and practice move to strengthen NHRS by member states.

#### Health Research legislation

Countries differed in their strategies to develop and deploy legislation for health research. Some countries had specific health research legislation; some embedded it in other overarching laws, while others used several instruments that are co-related. We however note the 17 out of 39 countries without a legislation on R4H and 2 out of 39 countries without ethical review committees.

### Enabling and constraining factors

Respondents highlighted the presence of good legal, governance and regulatory frameworks as key enabling factors. They also identified lack of functional and efficient coordination mechanism amongst government ministries and research institutions as major constraints to rapidly strengthen NHRS. Another identified constraint related to the affiliation of the research institutions. Where these were not government owned, they were reluctant to implement the government prioritised research agenda. Respondents also reported that there are few nationally and institutionally initiated and funded health research studies.

Although many countries had ethical review committees, the lengthy clearance process driven by bureaucracy was cited as a constraint. In addition, compliance with data protection laws was reported to delay commencement of research projects.

### Building and sustaining human, physical and institutional capacities on research for health

Significant increases were noted in the proportion of countries with a national health research institute/council rising from 59 to 72% over the review period. Noteworthy is the fact that 16 out of 39 countries lack a health research-promoting unit within the MOH while 11 out of 39 countries do not have a national health research institute/council.

The Regional average barometer score for developing and sustaining resources for R4H improved from 40 to 61% because of positive gains by all contributing indexes in this category (Table 4). The most improved indices in this category were the Regional universities with faculties of health sciences/medicine index (RUFHSI), Regional Research for Health programme index (RHRPRI) and the Regional NGO R4H index (RNGOI). Noteworthy were Kenya and Burkina Faso with a technical and support staff workforce in research programme of 1,365 and 500 respectively translating to a high staff density index. Under universities with faculties of health sciences/medicine index (RUFHSI), noteworthy was Ethiopia, South Africa, Kenya, Ghana and Angola who reported having substantial Universities that are conducting health research; Ethiopia (35), South Africa (24), Kenya (12), Ghana (10) and Angola (7).

### Enabling and constraining factors

Respondents identified the availability of multi-disciplinary experienced teams, young medical and bio-medical professionals keen on boosting research and, the motivation of quality research outputs of high impact on society and policy as some of the enabling factors for strengthening NHRS. However, key constraints identified were de-motivated staff, inadequate mechanisms for career progression and lack of experienced researchers. Additional constraints related to competing interests between conducting research & teaching and limited capacity to train researchers. The combination of brain drain and retirement of competent senior research scientists was noted as a double blow on maintaining a cohort of competent researchers on the continent. Furthermore, respondents cited the lack of infrastructure and equipment as constraints especially for undertaking biomedical and clinical research. In ameliorating research capacity challenges, respondents highlighted the role of partnerships in research with other universities, research consortiums as well as global partnerships to harness expertise and resources. However, in building partnerships, they cited the importance of trust and development of partnership frameworks for example Memorandum of Understanding (MoUs) to guide working arrangements.

### Producing and using research

Improvement was noted in the proportion of countries with Research and development (R&D) coordination mechanism in place which rose from 72 to 85% between 2014 and 2018, and consequently rapidly attaining the 2025 target. The proportion of countries with a knowledge translation platform increased from 41 to 59% although 16 out of 39 countries are yet to design and deploy such a platform. Knowledge translation platforms are discussion foras that bring together researchers and policy makers to discuss the available evidence, and together forge a plan of action to implement the recommendations from the evidence generated.

The Regional average barometer score for production and use of research improved from 35% in 2014 to 55% in 2018 even though data on Regional R4H publications per 100,000-population index (RPPCI) was not provided by all countries. The Regional knowledge translation platform index (RKTPI) increased significantly from 40 to 59% between the two surveys. The Regional health research management forum index (RHRMFI) only increased by 8% between the surveys.

Although there was an improvement in the score for the knowledge translation platform index, this area still poses significant challenges for member states. It is critical to link institutions, researchers, students, health policy makers and program managers to foster knowledge exchange and potentially influencing evidence-based policymaking. There were delays in use of research findings in planning and implementation.

### Financing of Reseach for health

The proportion of countries with a dedicated budget line for R4H increased from 51 to 62% between 2014 and 2018. The proportion of countries regularly tracking R4H spending from all sources increased by 8% over the same period (Table 3). Targeted financial investment in R4H is yet to be realised given the fact that 22 out of 24 countries with dedicated budget lines for research were yet to invest 2% of their national health budget in R4H and, 23 out of 24 countries were yet to invest 5% of their health sector development assistance in R4H.

The Regional average barometer index for financing R4H improved from 43% in 2014 to 61% in 2018, largely driven by countries creating a budget line for research. This index only represents 24 out of the 39 countries that submitted data on budget allocation.

**Fig. 1:**
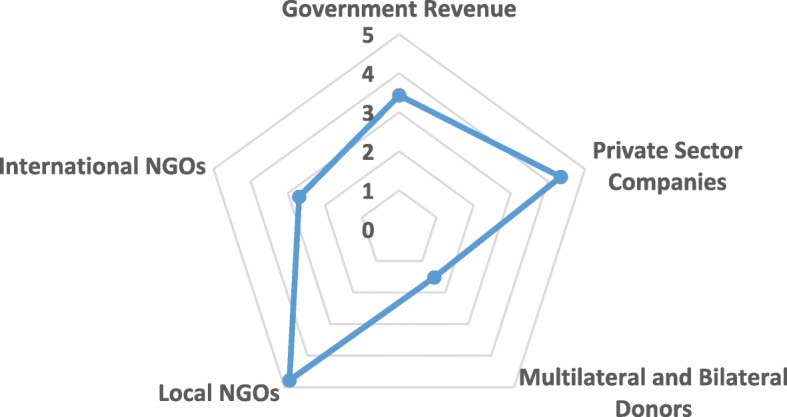
Health sector research financing and budget contribution ranked in order of decreasing importance

Respondents ranked on a scale of 1–6, the level of importance of the different sources of financing R4H with 1 being most important and 6 the least important. Figure 1, a radar graph derived from 23 out of 39 countries that responded to this particular question shows that countries ranked government third after multilateral and bilateral donors (first), and international NGOs (second) as sources of financing for R4H. The private sector ranked fourth and in the fifth place are local NGOs.

### Enabling and constraining factors

Domestic financing was identified as a key enabler for research for health. On the other hand, the key constraint reported was limited resource mobilisation skills among researchers resulting in low funding for research. Donor dependence in financing R4H, which responds to donor research interest and issues of global interest as opposed to local evidence needs, was cited as a challenge.

### Individual country scores

**Fig. 2:**
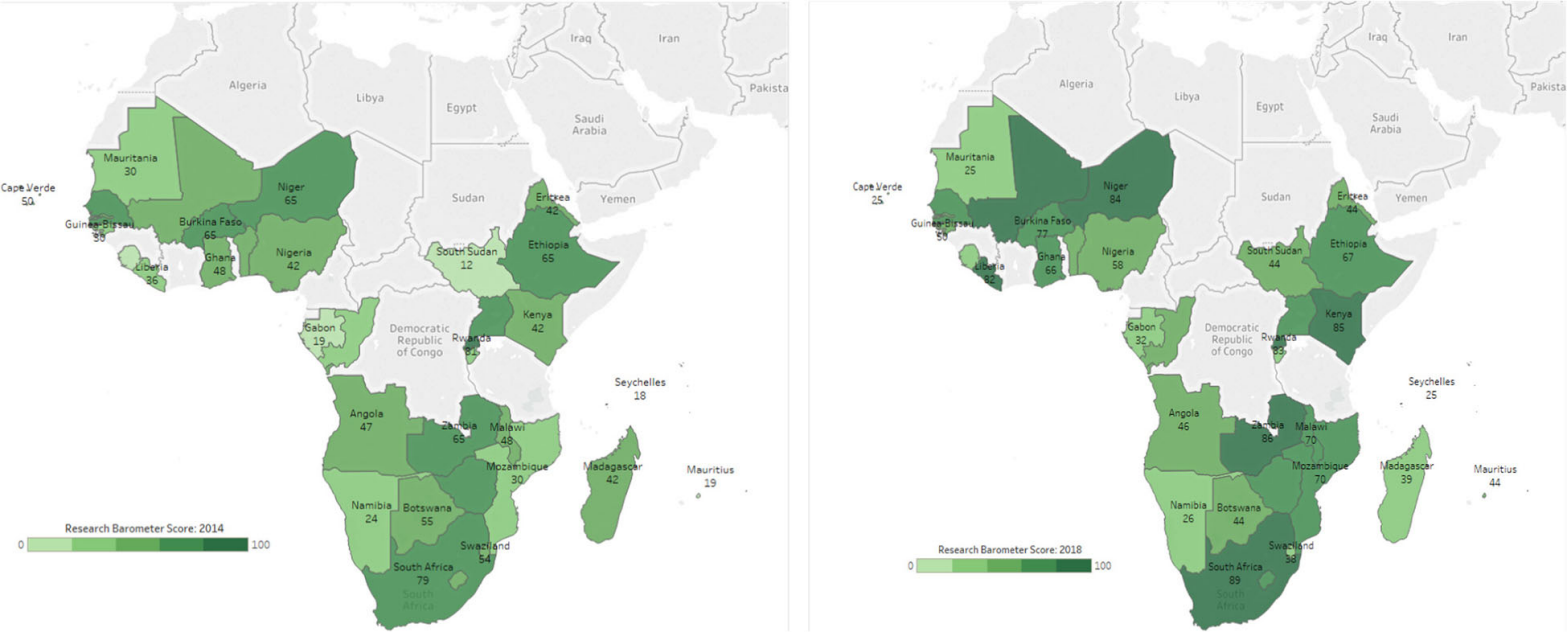
Individual country barometer scores

Country barometer scores for 2014 and 2018 were computed for the different countries as shown in Fig. 2. The following categorisation of barometers scores were employed to group countries: 0–19; 21–40; 41–60; 61–80 and 81–100. Noteworthy, there was no county in the category of 0–19 in the 2018 survey compared to 5 in the 2014 survey. We observed more countries in the 81–100 barometer score range in 2018 (9 countries) compared to 2014 (1 country).

### High and upper middle income countries

**Fig. 3:**
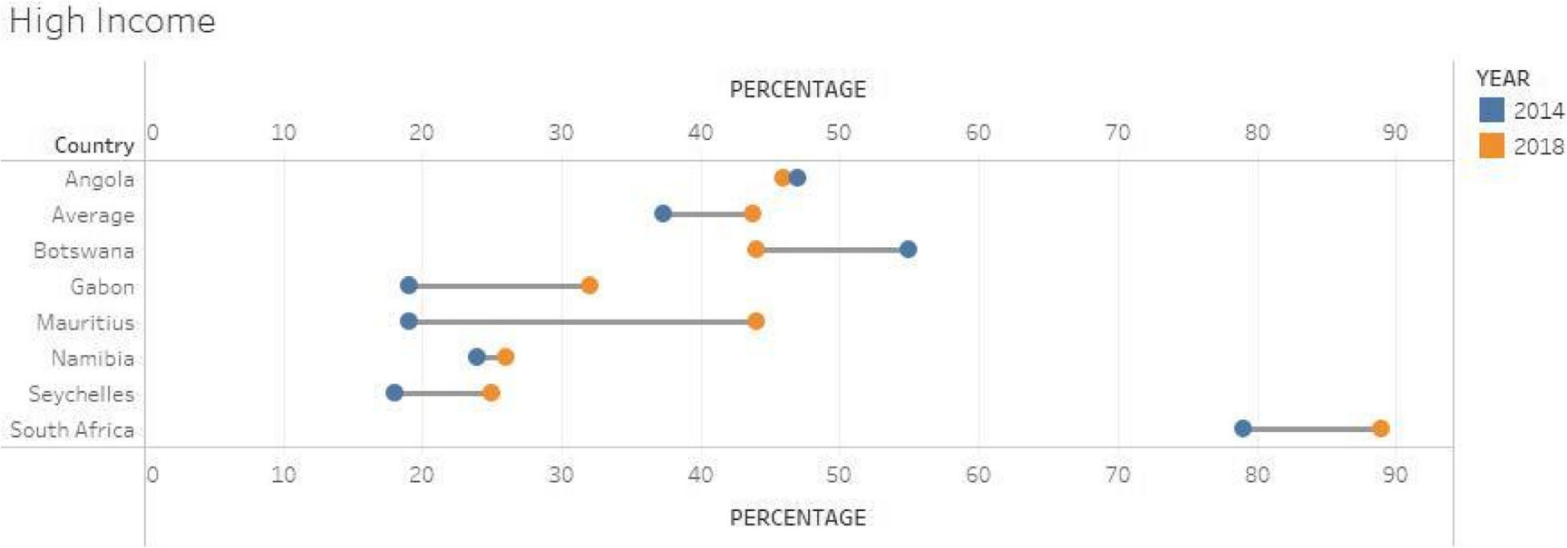
Group 1: Barometer scores for High income and upper middle income countries 2014 & 2018 (*n* = 7)

A total of 7 countries were in this category as shown in Fig. 3. The average score for this group of countries showed a 6%-point improvement between the two surveys. Of the 7 countries in this group, 5 showed improvements, whilst 2 showed some downward trend namely Angola and Botswana (although these countries did not submit all their budget allocations). Furthermore, for Botswana, the few technical and support staff (2) in research and only one university conducting health research led to the downward performance. In addition, the scores are very low for some countries (Namibia, Seychelles, Gabon) indicating very weak NHRS in these countries despite their level of income.

### Lower middle income countries

**Fig. 4:**
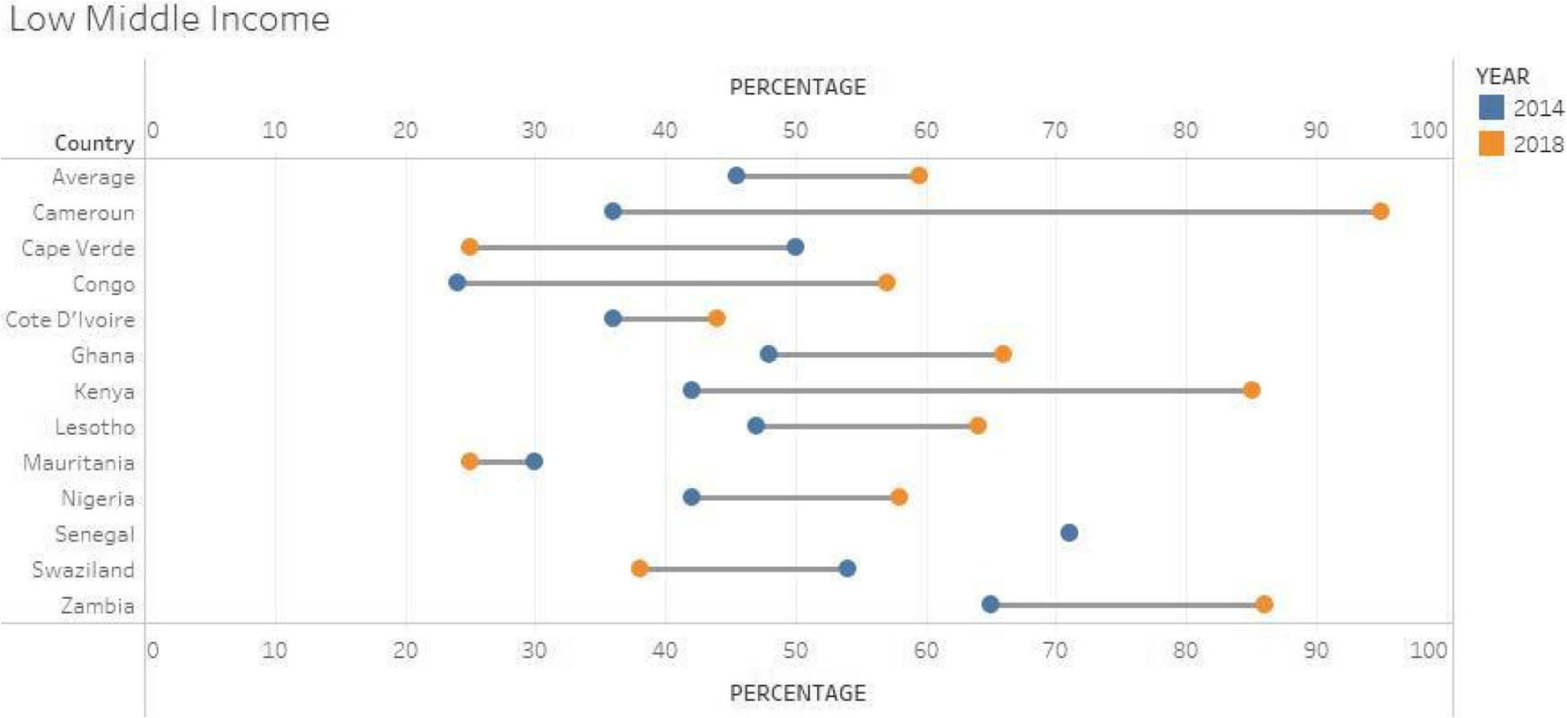
Barometer scores for Lower middle income countries 2014 & 2018 (*n* = 12)

Twelve countries were in this category as shown in Fig. 4. The average score for this group of countries showed a 14%-point improvement between the two surveys. In this category, 8 out of 12 countries showed improvements with the highest recorded for Cameroon, Kenya and Congo. Cameroon’s improvements were driven by significant budgetary allocations to health research, a fair number of universities conducting health research (6) and technical and support staff in health research programmes. Kenya’s technical and support staff in health research (1365) and universities conducting health research (12) contributed to the double increase of its overall index.

On the other hand, Cape Verde, Mauritania and Swaziland showed a decline in their overall performance indices. The lack of staff and universities conducting research (Cape Verde & Mauritania) as well budgetary allocations for research (Swaziland & Mauritania) contributed to the effect.

### Low income countries

**Fig. 5:**
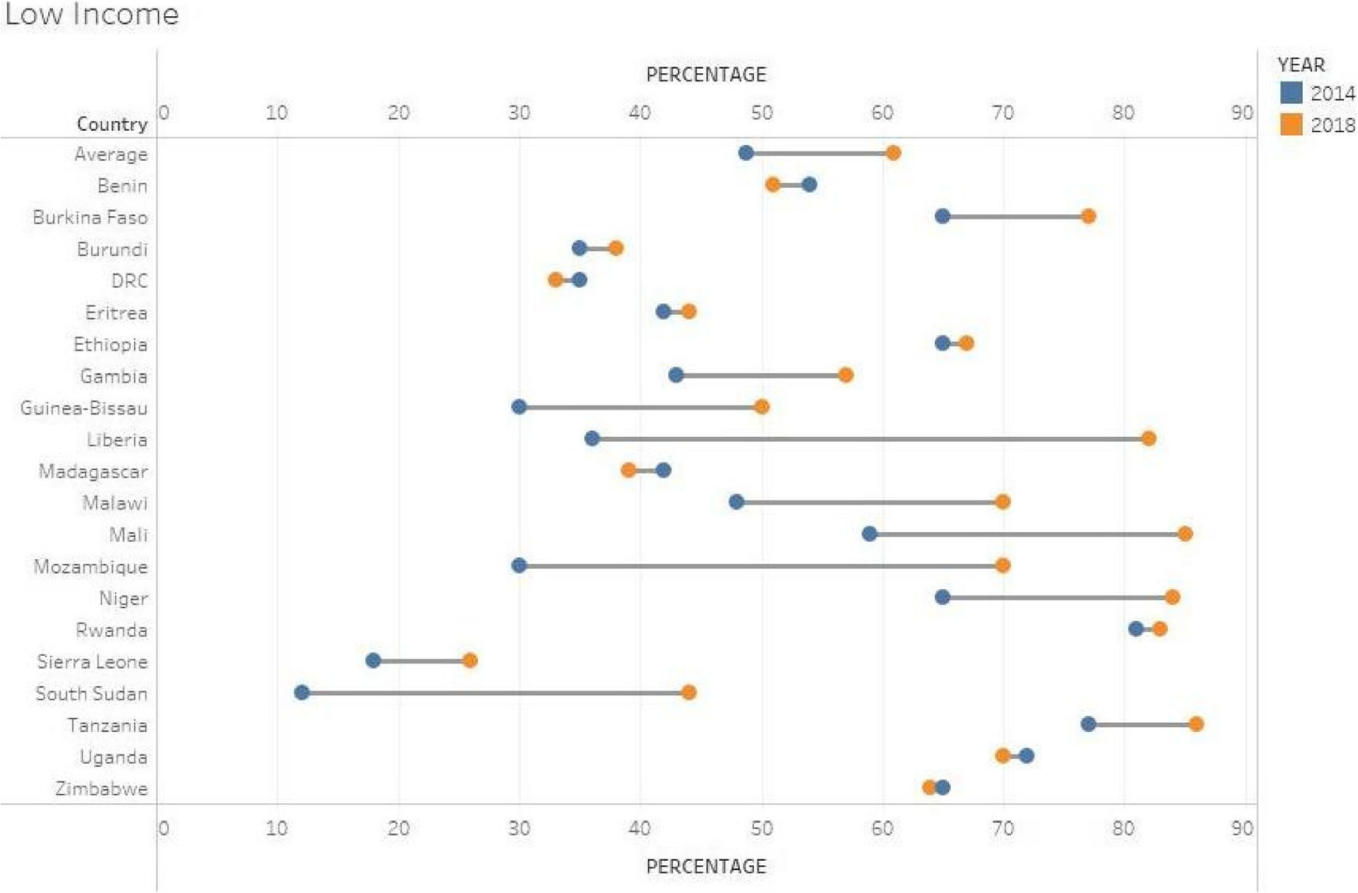
Barometer scores for Lower income countries, 2014 & 20 (*n* = 20)

Twenty countries were in this category as shown in Fig. 5. The average score for this group of countries showed a 12%-point improvement between the two surveys. Fifteen out of 20 countries showed significant improvements on their index. The improvements were mainly attributed to improvements on universities conducting health research and technical expertise in Liberia, Malawi and Mozambique whilst in Mali and Niger, budgetary allocations contributed more to their improved indexes.

Five countries showed some decline in their performance. These include Benin, DRC, Madagascar, Uganda and Zimbabwe. Lack of full budgetary allocations data for Benin, DRC and Zimbabwe were the main reasons for the drop in the indexes whilst few technical personnel to support health research and a low budgetary allocation to health research were some of the reasons for the index decline for Madagascar.

## Discussion

WHO AFR member states have made significant progress in strengthening their NHRS since the baseline assessment of 2014. Some of the indicators (4 out of 14) have either attained or exceeded the 2025 targets. The average Regional barometer score improved from 43% in 2014 to 61% in 2018. This indicates that African Region NHRS performance has improved from below average in 2014 to above average in 2018. There was improvement in almost all the barometer scores for all the NHRS sub functions. The performance could partially be explained by the guide provided by the WHO AFR research for health strategy adopted in 2014 at the Regional Committee. Least progress is seen under the financing for health research objective with stagnation in all indicators except one.

Identified constraints to strengthening NHRS include the weak research coordination mechanisms and weak linkages between government institutes that are involved in capacity building, undertaking and coordination of research. The low government financing for health and donor dependence is undermining efforts to build sustainable capacity for health research. The performance of NHRS varied within countries of the same level of economic development and between the different economic categories. A significant number of countries in the low income category had barometer scores that were higher than countries in the high and middle income categories.

### Governance for research

National governments as stewards of the country’s research agenda should set the strategic vision in policies and strategic plans, develop and enforce laws and regulations for research and protect research subjects through ethical processes. These serve to ensure that all resources and activities respond the country’s needs, local evidence gaps are addressed and research evidence supports the government’s development agenda as well as the UHC aspiration. Much progress has been realised in this area with regards to development of health research policies and strategies. But we also note the varied stages at which these strategic documents are in the different countries. While in some cases they are guiding implementation, in others they are expired but still under use or are stuck in protracted development processes. We did not assess the extent of implementation of the policies and strategies but the cited infrastructure, human and financial constraints, do impact implementation. Research laws and legislations were in place in only 56% of countries but even then, weak enforcement is a long standing problem. Umeakafor et al. (2016) [9] cite political influence, bribery and corruption among the hindrances to enforcing legislations. This notwithstanding, the status of these laws was varied, while in some countries they are stand-alone laws, in other cases they were embedded in other overarching laws for example the public health act, or multiple co-related instruments were in use which impacts on enforcement given the fact that the mandate to enforce these is housed in different ministries/institutions or, principles are articulated in policy documents which weakens their legal status.

Although national health research institutions are in place in many countries, the ownership of these has emerged as an issue in that, where they are not government owned, they do not focus on the government prioritised research agenda. We however argue that ownership of such institutions is not the bigger problem and instead we should focus on strengthening governance for research to foster collaboration whereby the institutions undertake research addressing questions identified by the government. This perhaps reflects the weak enforcement of the laws where they are in place or the lack of such in some countries that are yet to develop these. Additionally, 20 out of 39 countries in the 2018 survey did not have research strategies and prioritised research agendas and in such cases, there is no basis for aligning research institutions’ activities. Furthermore, the respondents attested to the lack of functional coordination mechanisms between government ministries and research institutions and this capacity must be built. Previous efforts have mainly focussed on governments coordinating donors’ activities and investment without paying much attention to coordination between government owned institutions. We however learn lessons from some countries (Ethiopia, Mali and Senegal) that have developed MoU between Ministry of health and health research institutions to guide engagements [10].

Significant progress has been realised in strengthening research ethics. Over the last decade, there has been significant investment towards strengthening of ethics and regulatory capacity in Africa. This has contributed to the improved NHRS through the work of different initiatives like the European and Developing Countries Clinical Trials Partnership (EDCTP), NIH Fogarty, Africa Medicines Harmonisation Initiative, Africa Vaccines regulators Forum (AVAREF) and others [11]. The lengthy clearance process, ethical review in emergency settings and compliance with data protection laws are the outstanding challenges. A lot of research for health tends to require stringent compliance with ethical requirements and data protection, it is important that governments, research institutions and universities train key staff in data management and protection, and streamline the processes involved whilst protecting patients and research participants adequately. There are positive lessons from Rwanda where a signing a memorandum of understanding to ensure confidentiality, intellectual property and data ownership is a mandatory requirement before any research commences.

### Creating and sustaining resources

The need for local solutions to attain UHC implies generation of contextually relevant evidence. In this regard, building local capacity to undertake research, manage and coordinate research processes is paramount. Our results show a positive trend in reference to training researchers and building capacity to conduct research. We look at these findings in two ways, where capacity has been built, there is need to ensure functionality which we did not assess in this study. However, when we look at the financing for research, our concern regarding functionality may be justified given the very low levels of funding. Only 2 countries are investing 2% of their national health budget into research. The low funding for research may undermine the investments in capacity building where researchers seek other opportunities due to lack of funds to undertake research. Indeed, demotivation, attrition and lack of career progression are highlighted as constraints to strengthening NHRS by respondents in our survey. Sitthi-amorn et al. (2000) note that the human resource constraints have contributed to limited capacity by developing countries to undertake research and use its results in policy development, as well as participation in political and global health debates [12].

Access to research funds especially on the international platform is very competitive and local scientists need to develop skills to write good project proposals. Hyder et al. (2003), in their study on doctoral training in Pakistan, found that only 2% of doctorate holders had more than two grants after training despite completion of their training 15 years earlier [13]. Another challenge that must be addressed is the competing interests between conducting research and teaching. Trostle et al. (1992) emphasized the need to create protected time for researchers in teaching institutions to undertake research [14]. Addressing this calls for innovation, one consideration would be to increase staff numbers but the human resource constraints in African countries is a hurdle to overcome. This impacts on the quality of research and perhaps partially explains the low volume of publications by African researchers.

Universal health coverage requires multi-dimensional interventions and multisectoral action and in this regard, the availability of multidisciplinary research teams as highlighted by the respondents is beneficial. However, the loss of competent researchers through retirement and attrition and the lack of research infrastructure and equipment present challenges. Respondents note the role of partnerships in ameliorating these challenges. Partnerships may or may not be beneficial depending on how they are negotiated and implemented. Chu et al. (2014) cautions us about exploitation in these partnerships citing divergent objectives, power imbalances and focusing on publication as opposed to skills transfer [15]. Another challenge is the short duration of such partnerships which does not allow enough time for building trust and transfer of skills [15]. Strengthening partnerships in research needs to be addressed at two levels, coordination at the national level to ensure alignment with national priorities and at the institutional level to ensure mutual benefits and skills transfer. Shortcomings at the institutional level have been cited as power imbalances and failure by African institutions to say “no” as such partnerships with western institutions are viewed as prestigious, and a potential source of income [16].

We note an increasing role of NGOs in undertaking research which is commendable but there is need to build their capacity in order to maximize their contribution to evidence generation and use. NGOs contribute to research processes and support uptake of evidence into policy and decision making but, their varied capacity in policy engagement, limited capacity to undertake research beyond their programmes, weak linkages with the researchers and donor dependency are major concerns [17].

### Producing and using research

The process of coordinating the research process (including paying attention to multisectoral approaches), using evidence in policy development and decision making and, increasing publications in peer reviewed journals by African scientists improved. More countries have put in place knowledge translation platforms which have been instrumental in development of evidence informed treatment policies [18], guidelines as well as policies [19]. We however highlight that getting evidence into policy takes more than having platforms in place, additional facilitating factors relate to the quality and timeliness of the evidence, effective dissemination and the implementation feasibility of the research recommendations [20]. Assessment of articles published by African researchers in peer reviewed journals was not assessed in the 2018 survey but had a very low barometer score in 2014. On the contrary, Hofman et al. (2009) in their study on mapping the trend of biomedical publications in MEDLINE by Africa authors showed an upward trend in the volume of publications although these were dominated by authors from South Africa, Nigeria and Kenya [21]. Uthman et al. (2015) showed a similar trend of growth in publication by African researches of 251% between 2000 to 2014, although as a share of the global research output, this increase translated into a growth of from 0.7 to 1.3% of the total global publications over the same period [22].

### Financing for research

The responsibility for building research capacity primarily lies with national governments and this must be reflected in making adequate investments. Surprising, in our survey respondents ranked government third as a source of funding for health research. Perhaps this derives from the current low level of government funding for health research. Our results show that budget lines have been created but this is not followed by allocating resources as only 2 countries are investing 2% of their national health budget in research. Does this mirror a low investment in health in general? Perhaps this is so because looking at the Abuja declaration [23] that committed African countries to allocate at least 15% of their national budget to health, we see a similar trend. Despite this declaration coming into force in 2001, by 2014 only 4 countries had met the target [23]. The low funding could be a reflection of lack of political will to invest in research. On other hand the low funding could be attributed to a low GDP and as such a limited fiscal space. This then calls for innovative ways to raise resources to fund health research. In this regard, there are examples countries can borrow from, for instance, the introduction of a tobacco tax (Togo & Carbo Verde) and alcohol tax and invested the revenues in health services, a proportion can be invested in research.

Efficient use of donor funds is another option, the major concern levied against this source of funding for research is the failure to address the country’s research priorities [24]. However, with good governance, enforcement of legislation and development of prioritized research agenda, donor funds can be used more effectively. In our survey, NGOs and the private sector are highlighted as potential sources of funding for research and perhaps these options need to be explored further. There are positive experiences but mainly from developed countries [13]. The low funding for research will undermine the investment in capacity building and strengthening governance for research. Wolffers et al. (1998) indeed cited the dependence of African research institutions on donor funding among the factors undermining sustainable capacity building for research [25].

### Country performance

Turning to individual country performance, we observed numerous variations and it is not clear what explains the performance of the NHRS. Within the high income category performance barometer scores ranged from a high 89% for South Africa to as low as 13% in Equatorial Guinea. In the low income category, performance ranged from as high as 83% for Rwanda to as low as 26% in Sierra Leone. We also noted that majority of low income countries were performing better than high income countries. This is contrary to the previous studies that showed a positive correlation between GDP, expenditure on R4H and human development index with health research publications [22, 26, 27]. Among the plausible explanations is the availability of research institutions (like the case of South Africa, Uganda, Kenya and Ghana) and funding for health research like the case of Cameroon that has allocated 2% of their health budget to research (with a barometer score of 85%) and South Africa that created a health research fund. Long term capacity building offers another explanation like the case of the 17 EDCTP supported countries that have benefitted from building capacity for ethics in research and infrastructure and skills to undertake clinical trials for a period of time. In this category of countries, 14 out of 17 registered improvement in their NHRS barometer scores between the two surveys and, all except 2 had barometers scores that were above average. Rwanda is another country that has built research capacity in a systematic manner over a period of time through well negotiated partnerships with western universities.

### Implications for policy and research

The NHRS has identified strengths and weaknesses of NHRS within the WHO AFR member states. In order to realise functional NHRS, all the four objectives of the Regional health research strategy must be strengthened namely: Governance of research for health, Developing and sustaining resources for R4H, Producing and using R4H, and Financing of R4H. The findings of this assessment provide evidence to inform the development of health research policy and strategic plan to address identified gaps as well as consolidations of gains. These strategic documents should articulate the vision and goal for health research in the country, priority interventions, implementation arrangements, roles and responsibilities of stakeholders and a monitoring framework. These should guide resource mobilisation and allocation decisions. Laws and legislations must be developed where lacking and enforced.

Countries should endeavour to build a system for health research taking into account the different components of systems. These include, mobilizing inputs (human, financial, institutions, infrastructure, tools and guidance documents), undertaking processes (implementation of interventions, capacity building), producing outputs (using inputs to undertake processes, e.g. researchers trained), outcomes (capacity built, timely production of evidence, publication in peer reviewed journals) and impact (strengthened NHRS). There is need to intervene in all the components of the system in order to realise strengthened NHRS. This should be backed by strong monitoring to assess progress.

### Study limitations

The NHRS barometer methodology we employed focusses more on the presence or the absence thereof of the different sub functions under the different objectives of the regional research for health strategy. In order to strengthen NHRS, it takes more than availability of policies and strategies, presence of research institutes, research coordination mechanisms etc. These must be implemented, laws enforced and mechanisms functional. The NHRS barometer methodology needs to be refined to incorporate a functional component.

## Conclusion

African countries have made strides in building research capacity as evidenced by the significant improvement in the NHRS indices. While the indices show improved overall performance, some countries are still below the target and need to close the identified country-specific gaps. Furthermore, the study provides an opportunity for countries to share best practice in areas of excellence. In responding to these findings, domestic financing will be crucial to realising sustainable progress in strengthening NHRS for the generation of local and contextualised solutions towards UHC. A case has been made for exploring innovative mechanisms as well as the role of private entities in funding local research. In addition, creating balanced, trustworthy and responsible partnerships and collaborations, and avoiding working in silos for research centres, hospitals/health centres, academic institutions, as well as government departments involved in research activities within a country can help countries strengthen their NHRS and bear fruit in terms of better health services for the citizens.

Until countries develop sustainable financing mechanisms for health research, funding from international sources definitely will continue to play a role, but strong governance to ensure coordinated efforts and alignment to country priorities will be key to attaining maximum return on investment.

## Data Availability

Please contact the authorship team to enquire regarding access to material.
